# Putrescine as a Novel Biomarker of Maternal Serum in First Trimester for the Prediction of Gestational Diabetes Mellitus: A Nested Case-Control Study

**DOI:** 10.3389/fendo.2021.759893

**Published:** 2021-12-14

**Authors:** Cheng Liu, Yuanyuan Wang, Wei Zheng, Jia Wang, Ya Zhang, Wei Song, Aili Wang, Xu Ma, Guanghui Li

**Affiliations:** ^1^ Division of Endocrinology and Metabolism, Department of Obstetrics, Beijing Obstetrics and Gynecology Hospital, Capital Medical University, Beijing Maternal and Child Health Care Hospital, Beijing, China; ^2^ National Research Institute for Family Planning, Beijing, China; ^3^ National Human Genetic Resources Center, Beijing, China

**Keywords:** putrescine, gestational diabetes mellitus, biomarker, prediction, receiver operating characteristic (ROC)

## Abstract

**Aims:**

Early identification of gestational diabetes mellitus (GDM) aims to reduce the risk of adverse maternal and perinatal outcomes. Currently, no acknowledged biomarker has proven clinically useful for the accurate prediction of GDM. In this study, we tested whether serum putrescine level changed in the first trimester and could improve the prediction of GDM.

**Methods:**

This study is a nested case-control study conducted in Beijing Obstetrics and Gynecology Hospital. We examined serum putrescine at 8-12 weeks pregnancy in 47 women with GDM and 47 age- and body mass index (BMI)-matched normoglycaemic women. Anthropometric, clinical and laboratory variables were obtained during the same period. The receiver operating characteristic (ROC) curve and area under the curve (AUC) were used to assess the discrimination and calibration of the prediction models.

**Results:**

Serum putrescine in the first trimester was significantly higher in women who later developed GDM. When using putrescine alone to predict the risk of GDM, the AUC of the nomogram was 0.904 (sensitivity of 100% and specificity of 83%, 95% CI=0.832–0.976, P<0.001). When combined with traditional risk factors (prepregnant BMI and fasting blood glucose), the AUC was 0.951 (sensitivity of 89.4% and specificity of 91.5%, 95% CI=0.906-0.995, P<0.001).

**Conclusion:**

This study revealed that GDM women had an elevated level of serum putrescine in the first trimester. Circulating putrescine may serve as a valuable predictive biomarker for GDM.

## Introduction

Gestational diabetes mellitus (GDM), a common gestational disorder, has been defined as glucose intolerance with onset or first detection during the second or third trimester (1). GDM has short and long-term adverse effects on the health of the mother and their offspring. Women with GDM are more likely to have preeclampsia, caesarean section and are likely to have a higher rate of developing type 2 diabetes mellitus (T2DM) and cardiovascular disease in the postpartum period than those without GDM (2–4). For neonates, GDM increases the risk of macrosomia, hypoglycaemia, shoulder dystocia, and respiratory distress syndrome (5). Additionally, the offspring of GDM women are more likely to develop obesity and abnormal blood glycaemia in later life (6). Currently, the diagnosis of GDM is based on the oral glucose tolerance test (OGTT) which is generally performed in the second trimester of the pregnancy. Although there is no consensus about screening algorithms and diagnostic criteria for GDM before 24 weeks of gestation, risk stratification in the first trimester could be beneficial to reduce adverse complications associated with GDM due to timely and targeted lifestyle interventions such as physical exercise and dietary changes (7). Thus, establishing a simple, practical prediction model of GDM according to the identified risk factors in the first trimester is of great clinical significance. However, the present predictive biomarker and model of GDM did not show a pronounced and reliable predictive value (8).

Putrescine is one of the predominant polyamine in mammalian cells and is pervasive in a wide range of organisms because it is necessary for cell growth and proliferation (9). Disturbance of the gut microbiota can cause an imbalance in polyamine metabolism, which may be related to the pathological development of metabolic diseases (10, 11). In our former study, we had reported that women with GDM already had different components of gut microbiota than normoglycaemic women in the first trimester (12). Through the Kyoto Encyclopedia of Genes and Genomes (KEGG) annotation and pathway enrichment analysis, we correlated multiple small molecular metabolites with gut microbiota that enriched in GDM. We found that seven intestinal bacteria enriched in GDM were significantly correlated with the metabolism of putrescine. Furthermore, putrescine has recently been shown to associate with the disrupted tight junction (TJ) integrity in the colon (13). It could decrease the intestinal mucosal barrier function and increase bacterial translocation, contributing to the onset of systemic inflammation-associated metabolic disorders. A recent case-control study found that serum putrescine levels in patients with T2DM were significantly higher than those in the control group, and abnormally elevated putrescine levels were related to insulin resistance and glycosylated haemoglobin levels (14). However, the relationship between serum putrescine levels and GDM has not yet been reported.

To the best of our knowledge, no study has evaluated the relationship between putrescine and GDM risk. Based on the findings in the GDM gut microbiota and the relationship between putrescine and the intestinal barrier, we hypothesized that serum putrescine levels might be associated with the development of GDM. If so, putrescine may serve as an effective predictive biomarker for GDM.

## Research Design and Methods

### Patient Cohorts

The participants in this nested case-control study were from a prospective cohort study in the Beijing Obstetrics and Gynecology Hospital, Capital Medical University. All pregnant women who intended to give birth in this hospital were enrolled in the cohort study at 8–12 weeks of gestation and followed until delivery.

To evaluate the relationship between serum putrescine and GDM, we selected eligible subjects from the recruited pregnant women above. Women of 18 to 44 years of age and with a singleton pregnancy were recruited and only participants with complete clinical information were included in the analysis. Subjects were excluded if they had pre-existing chronic medical conditions, including hypertension, T2DM, and heart or kidney diseases. Pregnant women diagnosed with diabetes or impaired glucose tolerance in the first trimester were also excluded. GDM was diagnosed at gestational week 24 to week 28 according to American Diabetes Association (ADA) criteria (15), and a 75-g OGTT was performed to screen for GDM. Women with GDM were enrolled when any of the following criteria were met: fasting plasma glucose (FBG) ≥ 5.1 mmol/L, plasma glucose at 1 h ≥ 10 mmol/L, or plasma glucose at 2 h ≥8.5 mmol/L. Normoglycaemic women were matched for age ( ± 3 years) to each case of GDM women in the same cohort. We also matched the pre-pregnancy BMI according to the classification of Institute of Medicine (IOM) (16). The study was approved by the Ethics Committee of Beijing Obstetrics and Gynecology Hospital (2017-KY-015-01). Written informed consent was obtained from every participant. All procedures were conducted according to the guidelines laid down in the Declaration of Helsinki.

### Clinical Measurements and Covariates

Anthropometric measurements of participants were completed by trained medical staff at recruitment using a standardized protocol. Clinical data were collected by medical record review. Pre-pregnancy body weight was self-reported. A family history of diabetes was defined as a first-degree relative with T2DM. Smoking was defined as either ongoing smoking or former smoking and never smoking (No). Drinking was categorized as never/occasional (No) and regular. The FBG and lipid profiles, including cholesterol (CHOL), triglyceride (TG), high-density lipoprotein (HDL), and low-density lipoprotein (LDL), were determined as described in a previous study (17).

### Putrescine Examination

Blood samples were collected from participants following an overnight fast at 8–12 weeks, and serum specimens were isolated and stored at -80°C for further examination. The serum putrescine levels were examined by liquid chromatography coupled to tandem mass spectrometry (LC-MS/MS, Thermo Scientific, USA). First, 100 μL of human serum was briefly added to a 0.5 mL glass centrifuge tube. After centrifugation at 14000 r/min for 5 min, the serum sample was dried under nitrogen at 50°C. Then, 60 μL of n-butyl alcohol and 12 mol/L HCI (95:5 v/v) were added and vortexed for 30 seconds in a seal. After incubation at 65°C for 15 min for derivatization, the derivatized solution was centrifuged, and dried under nitrogen at 50°C again. The residue was reconstituted by adding 100 μL of acetonitrile and water (4:1, v/v), vortexed for 30 seconds, centrifuged at 14000 r/min for 5 min and injected at 20 μL for LC-MS/MS analysis.

An API 4500 Qtrap (Agilent 1260 LC equipped with an ESI ion source) was employed for the analysis. Putrescine was separated on an FFAP elastic quartz capillary column (30 m× 0.25 mm× 0.25 μm) from interfering substances in the matrix. Mobile phases consisted of 80% acetonitrile (containing 0.1% formic acid). The pump flow rate setting is variable: 140 μL/min × 0.2 min, 30 μL/min × 1. 3 min and 300 μL/min × 0.5 min. The automatic injection device was set to inject 20 μL each time, and the sampling needle was flushed before injection. The chromatographic column temperature programme: initial temperature 60°C, 0-8 min increased to 180°C, retention for 2 min; carrier gas: high-purity nitrogen (purity 99.999%); carrier gas flow rate 0.89 ml/min; inlet temperature: 200°C. Data were acquired in multiple reaction monitoring (MRM) mode.

### Sample Size and Statistics

The sample size was calculated using MedCalc v20.0.3. The total sample size was 94 when set the true proportion as 0.70 and the null hypothesis proportion as 0.50, and hypothesize α=0.01, β=0.20. Data were analysed using the SPSS 22.0 software. Data with normal distributions are shown as the mean ± standard deviation, and nonnormal distributed data are shown as the median (interquartile range), respectively. T-tests and Wilcoxon tests were used to analyse the differences in continuous variables between the GDM group and the control group. Serum putrescine concentrations were also compared by t-test. Categorical variables, including serum putrescine levels (categorized into quartiles), were evaluated using the X^2^ test or Fishers’s exact test. Although women in the control group were matched for age and prepregnancy BMI to each case of GDM women in the same cohort, the prepregnancy BMI was still higher in the GDM group. Thus, we adjusted the prepregnancy BMI when comparing the serum level of putrescine between the two groups. Binary logistic regression for the association between GDM and serum putrescine was carried out with adjustment for potentially confounding variables. The results are represented by the odds ratio (OR) and 95% confidence interval (CI). To further specify the clinical significance of putrescine, we built 3 multivariate predictive models using putrescine with or without traditional risk factors (i.e., maternal age, prepregnancy BMI, history of family diabetes, FBG and lipid profile) and the traditional risk factors only to evaluate the risk of GDM. The receiver operating characteristic (ROC) curve analysis was performed, and the area under the curve (AUC) was calculated to evaluate the accuracy, sensitivity and specificity of the model. The differences were considered statistically significant when P<0.05.

## Results

### Clinical and Laboratory Characteristics

This study comprised 47 GDM women and 47 normoglycaemic controls. Although we matched the two groups according to age and BMI, the prepregnancy BMI was significantly higher in the GDM group than in the control group. As shown in **Table 1**, there were no statistically significant differences in other clinical characteristics (P>0.05). However, the lipid and glucose parameters in the first trimester were significantly different between the two groups. Serum lipid parameters, including CHOL, TG, HDL, and FBG were significantly higher in the GDM group (P<0.05).

**Table 1:** Anthropometric, clinical and laboratory variables in women with GDM and controls.

	GDM (N = 47)	Control (N = 47)	P
Age	33.0 ± 3.57	32.1 ± 2.88	0.161
Height	162.04 ± 4.98	163.82 ± 5.78	0.118
Pre-pregnancy weight	58.65 ± 7.84	55.60 ± 7.03	0.053
Pre-pregnancy BMI	22.32 ± 2.69	20.68 ± 2.01	0.001**
Gravidity	1.98 ± 1.14	1.74 ± 0.96	0.278
Parity	1.38 ± 0.53	1.32 ± 0.47	0.532
Family history of diabetes			0.284
Yes	11 (23.4%)	6 (12.8%)	
No	36 (76.6%)	41 (87.2%)	
Smoking			1
Yes	2 (4.3%)	1 (2.1%)	
No	45 (95.7%)	46 (97.9%)	
Drinking			1
Yes	5 (10.6%)	4 (8.5%)	
No	42 (89.4%)	43 (91.5%)	
Education background			1
University or above	38 (80.9%)	39 (83.0%)	
Junior college or below	9 (19.1%)	8 (17.0%)	
Laboratory parameter			
FBG (mmol/L)	4.86 ± 0.49	4.64 ± 0.36	0.015*
CHOL (mmol/L)	4.46 ± 0.73	4.12 ± 0.64	0.019*
TG (mmol/L)	1.26 (0.91-1.63)	1.02 (0.85-1.26)	0.023*
HDL (mmol/L)	1.48 ± 0.30	1.49 ± 0.29	0.920
LDL (mmol/L)	2.33 ± 0.60	2.08 ± 0.47	0.026*
Putrescine (μmol/L)	52.10 (48.00-55.12)	33.16 (20.09-39.74)	<0.001***

*P < 0.05. **P < 0.01. ***P < 0.001.

GDM, gestational diabetes mellitus; BMI, body mass index; FPG, fasting plasma glucose; CHOL, cholesterol; TG, triglyceride; HDL, high density lipoprotein; LDL, low density lipoprotein.

### Serum Putrescine Levels Between GDM and Normoglycemic Women

**Figure 1:**
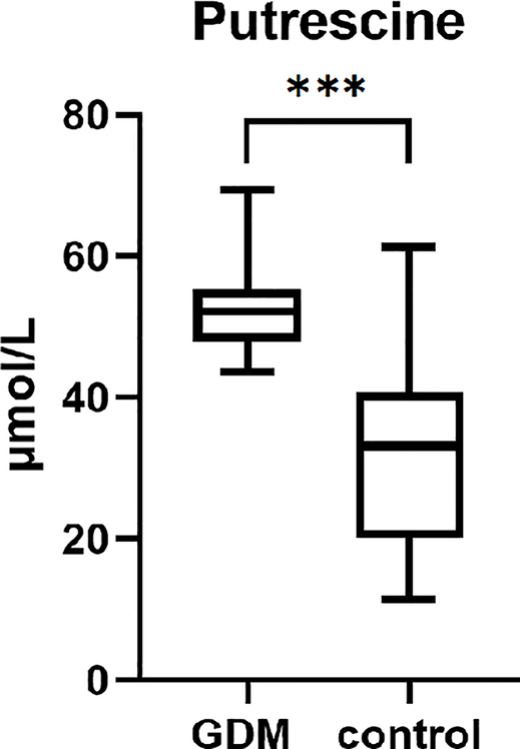
The serum putrescien in the first trimester of women with GDM and controls. (***P < 0.001).

The serum putrescine concentration was significantly higher in women with GDM than in women compared to the controls in the first trimester. After adjusted the prepregnancy BMI, the difference was still significant (P<0.001, **Figure 1**). When stratified by quartile, those in the upper quartile of putrescine concentration were at increased risk of GDM compared to those with the lowest putrescine concentration (**Table 2**).

**Table 2:** The quartile stratification comparison of putrescine.

	GDM (N = 47)	Control (N = 47)	X^2^	P
Putrescine (μmol/L)			50.80	<0.001***
<33.36	0	24		
33.36-45.07	7	16		
45.08-52.70	19	4		
>52.70	21	3		

***P < 0.001.

GDM, gestational diabetes mellitus.

### Traditional Risk Factors for GDM Explored by Logistic Regression

Regarding clinical risk factors (**Table 3**), univariable logistic regression showed that only prepregnancy BMI was positively correlated with GDM risk (P<0.05). However, a family history of diabetes and smoking may increase the risk of GDM, with an ORs of 2.088 (95% CI = 0.701-6.215) and 2.044 (95% CI = 0.179-23.348) respectively. Regarding laboratory risk factors (**Table 3**), FBG, CHOL, TG and LDL in the first trimester were significantly and positively correlated with GDM risk (P<0.05).

**Table 3:** Univariate logistic regression for GDM.

	B	P	OR (95% CI)
Age	0.089	0.169	1.093 (0.963-1.240)
Height	-0.061	0.120	0.941 (0.871-1.016)
Pre-pregnancy weight	0.055	0.056	1.056 (0.999-1.117)
Pre-pregnancy BMI	0.288	0.003**	1.333 (1.106-1.608)
gravidity	0.213	0.287	1.237 (0.836-1.829)
parity	0.257	0.536	1.293 (0.573-2.919)
family history of diabetes	0.736	0.186	2.088 (0.701-6.215)
smoking	0.715	0.565	2.044 (0.179-23.348)
Drinking	0.247	0.726	1.280 (0.321-5.096)
Education background	0.144	0.789	1.155 (0.403-3.306)
Laboratory parameter			
FBG	1.393	0.022*	4.027 (1.221-13.283)
CHOL	0.710	0.025*	2.035 (1.092-3.793)
TG	1.002	0.022*	2.723 (1.154-6.427)
HDL	-0.070	0.921	0.932 (0.233-3.733)
LDL	0.880	0.030*	2.412 (1.089-5.343)
Putrescine	0.208	<0.001***	1.231 (1.130-1.341)

*P < 0.05. **P < 0.01. ***P < 0.001.

GDM, gestational diabetes mellitus; BMI, body mass index; FPG, fasting plasma glucose; CHOL, cholesterol; TG, triglyceride; HDL, high density lipoprotein; LDL, low density lipoprotein.

**Figure 2:**
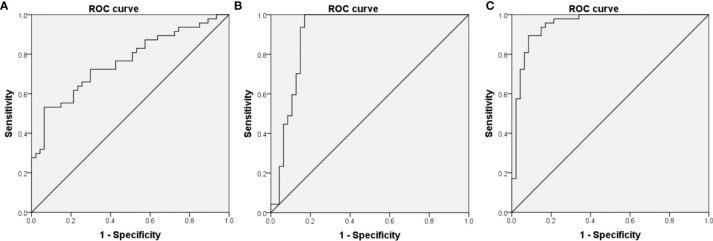
Receiver operating characteristic (ROC) curves for logistic regression models utilizing clinical risk factors **(A)**, serum putrescine **(B)** serum putrescine and clinical risk factors **(C)**.

According to the univariable logistic regression, we used prepregnancy weight, prepregnancy BMI, family history of diabetes, smoking, FBG, CHOL, TG and LDL to build a traditional risk prediction model. An ROC curve was constructed using the model with an AUC of 0.761 (sensitivity of 53.2% and specificity of 93.6%, 95% CI=0.665–0.858, P<0.001, **Figure 2A**).

### The Prediction Effect of Serum Putrescine for GDM

Univariable logistic analyses indicated that serum putrescine concentration was significantly and positively associated with a higher risk of GDM (OR=1.231, 95% CI=1.130–1.341, P<0.001). After adjustment for maternal age, prepregnancy BMI, history of family diabetes, FBG and lipid profile, the results did not change (OR=1.274, 95% CI=1.148–1.413, P<0.001).

To evaluate the performance of serum putrescine as predictive biomarker for GDM, ROC curves were constructed using the putrescine with an AUC of 0.904 (sensitivity of 100% and specificity of 83%, 95% CI=0.832–0.976, P<0.001, **Figure 2B**). When putrescine was combined with traditional risk factors, the final risk prediction model included prepregnancy BMI, FBG and putrescine (**Table 4**), which achieved an AUC of 0.951 (sensitivity of 89.4% and specificity of 91.5%, 95% CI=0.906-0.995, P<0.001, **Figure 2C**).

**Table 4:** Multivariate logistic regression for GDM.

	B	P	OR (95% CI)
Putrescine	0.242	<0.001***	1.274 (1.148-1.413)
Pre-pregnancy BMI	0.441	0.009**	1.555 (1.118-2.162)
FBG	2.102	0.051	8.183 (0.990-67.642)
constant	-30.123	<0.001***	0

**P < 0.01. ***P < 0.001

GDM, gestational diabetes mellitus; BMI, body mass index; FPG, fasting plasma glucose.

## Discussion

Our study demonstrated that serum putrescine levels in the first trimester were significantly higher in women who later developed GDM. Measurement of serum putrescine concentration showed excellent diagnostic value for predicting the risk of GDM. We also built a final model that included putrescine and traditional clinical risk factors (prepregnancy BMI and FBG). And this model showed a considerable degree of discrimination and achieved an AUC of 0.951.

There is evidence that some women with GDM already have abnormal glucose metabolism in the first trimester (18). However, the diagnosis of GDM is often made during the second trimester, which presents a limited time for an intervention. Detection of women at risk earlier during pregnancy would be important to enable early lifestyle modification or even drug treatment to improve the perinatal outcomes of these women. Under those circumstances, using new risk prediction models to improve the existing selective screening algorithms and therefore to effectively reduce the number of inconvenient and belated oral glucose tolerance tests is meritorious.

There are many traditional risk factors for GDM, including advanced maternal age, overweight or obesity, ethnicity, family history of diabetes, history of GDM in a previous pregnancy, high FBG, glycated haemoglobin A1c (HbA1c) and lipid profile. However, the predictive performance of these risk factors is criticized as having limited diagnostic accuracy (8, 19, 20). The AUC values of these traditional clinical variables ranged from 0.6-0.8, and most of these previous studies used different GDM diagnosis criteria (8, 21, 22). Therefore, their clinical benefit when applied to the most recent GDM definition has not yet been well investigated. Recently, a number of new markers have been evaluated for use in predicting GDM with variable success, such as protein biomarkers (23), adiponectin (24) and leptin (25), pentraxin 3 (26), trace elements (27), RNA (28), single-nucleotide polymorphisms (29), and other combined metabolite models (30–33). The AUC values of some of these models can reach above 0.8. However, interindividual variability, sample collection and transportation, confounding variables and cost limit the application of these new markers in clinical.

Metabolomics is a rapidly developing science that aims to quantitatively describe the dynamic changes of many metabolites in organisms. Metabolomics has the capacity to recognize early deregulations and disruptions in metabolism associated with diseases or disorders. To investigate physiological processes and to develop early diagnostics, metabolomics is one of the most promising technologies (34, 35). Plasma metabolomics is a powerful technology for the rapid and profound analysis of all kinds of metabolic diseases. The amount of information obtained by this approach is still not fully understood. The GDM prediction model from the UK Pregnancies Better Eating and Activity Trial (UPBEAT), which included multiple serum metabolites, reached an AUC of 0.78 (30). Other metabolite models that included adiponectin reached an AUC of 0.79-0.85 (24, 33). A study using untargeted and targeted metabolomic protocols to analyse plasma and urine samples of pregnant women with and without GDM reported that the combination of 11 metabolites from blood samples and 5 metabolites from urine samples improved the AUC prediction accuracy to 0.99 (31). However, this study only investigated 14 GDM women and 18 non-GDM women. The multivariable predictive model based on a small sample size may have limited promotion value. In addition, the association between metabolites and the risk of a certain disease may considerably vary among different ethnicities and the heterogeneity of GDM. Alternately, different GDM diagnosis criteria across studies and the diversity of different laboratory instruments may also influence the effects of prediction and extrapolation. Although combined clinical risk factors and metabolite biomarkers improve the prediction of GDM, selective screening based on the presence of one or more risk factors has shown to have limited diagnostic accuracy (36, 37). In light of these previous studies, the model in this study included only one metabolite, which can reach an AUC of 0.95 and a sensitivity of 89.4% is encouraging.

Some studies reported that elevated serum putrescine levels were associated with T2DM (14). However, there are few reports about putrescine metabolism in pregnant women with GDM. Putrescine is a metabolite of intestinal bacteria and is produced by collective biosynthetic pathways of the commensal microbiome (38). In recent years, the gut microbiota and its metabolites have shown a significant relationship with GDM. Our former study demonstrated that women who develop GDM exhibit distinct gut microbiota compositions in the first trimester, and the bacteria enriched in GDM have been found to be related to the metabolism of putrescine (12). A recent study conducted high-throughput sequencing of thousands of indicators, including secreted proteins, microbial metabolites, and drugs, and found for the first time that putrescine can destroy intestinal barrier function by disrupting TJ integrity and cause colon inflammation (13). Disruption of gut barrier integrity generates a “leaky gut”, allowing the influx of bacterial ligands and inflammatory cytokines into the portal and systemic circulation through leaky gut to trigger systemic inflammation in a broad range of target organs, such as adipose tissues. GDM, similar to T2DM, is an inflammatory clinical entity with different mechanisms involved in its physiopathology. Impaired intestinal barrier structure and function are important to the low-grade inflammation (39) and have been validated as an important pathogenic process in T2DM (40, 41). Chronic systemic inflammation and adipose tissue inflammation have also been regarded to play a role in the progression of GDM (42, 43). This may suggest the pathogenic mechanism of putrescine, which impairs the intestinal barrier and triggers chronic inflammation, in GDM. Although the direct cause and effect relationship between putrescine and its pathological states has not yet been confirmed, reversing harmful intestinal microbiota metabolites may become an attractive and potent target for disease prevention and treatment.

Our current study revealed that women with GDM had an elevated level of serum putrescine in the first trimester. We first used serum putrescine levels in the first trimester to build a sensitive and reliable prediction model of GDM, which could help identify high-risk individuals at an early stage. However, this study was a single-center study, limited by the sample size and restricted ethnicity. Furthermore, the metabolome is influenced by patient intrinsic factors such as ethnicity, epigenetics, and genetic mutations, and extrinsic factors such as environment, stress, and diet. We did not conduct the diet survey and stress assessment, so we were unable to evaluate these extrinsic factors. This conclusion deserves to be verified in a large and multiethnic population cohort and the influencing factors of metabolome should be evaluated. The causality of putrescine and GDM may be connected by disruption of the intestinal barrier and systemic and local inflammation. The specific mechanism between putrescine and GDM requires to be explored in greater depth.

## Data Availability Statement

The raw data supporting the conclusions of this article will be made available by the authors, without undue reservation.

## Ethics Statement

The studies involving human participants were reviewed and approved by Ethics Committee of Beijing Obstetrics and Gynaecology Hospital. The patients/participants provided their written informed consent to participate in this study.

## Author Contributions

GL and XM contributed to the study design and interpretation of the data. CL and YW contributed to the drafting and revision of the manuscript. CL and WZ coordinated and executed the statistical analysis. JW and YZ contributed to the collection of data. AW and WS contributed to the enrollment and follow-up in clinic. All authors reviewed and approved the final, submitted version.

## Funding

This work was supported by The National Key Research and Development Program of China (2016YFC1000304), Beijing Hospitals Authority’ Ascent Plan (DFL20191402).

## Conflict of Interest

The authors declare that the research was conducted in the absence of any commercial or financial relationships that could be construed as a potential conflict of interest.

## Publisher’s Note

All claims expressed in this article are solely those of the authors and do not necessarily represent those of their affiliated organizations, or those of the publisher, the editors and the reviewers. Any product that may be evaluated in this article, or claim that may be made by its manufacturer, is not guaranteed or endorsed by the publisher.
